# Allometric scaling in-vitro

**DOI:** 10.1038/srep42113

**Published:** 2017-02-07

**Authors:** Arti Ahluwalia

**Affiliations:** 1 Department of Information Engineering and Research Center E.Piaggio, University of Pisa, Pisa, Italy

## Abstract

About two decades ago, West and coworkers established a model which predicts that metabolic rate follows a three quarter power relationship with the mass of an organism, based on the premise that tissues are supplied nutrients through a fractal distribution network. Quarter power scaling is widely considered a universal law of biology and it is generally accepted that were *in*-*vitro* cultures to obey allometric metabolic scaling, they would have more predictive potential and could, for instance, provide a viable substitute for animals in research. This paper outlines a theoretical and computational framework for establishing quarter power scaling in three-dimensional spherical constructs *in*-*vitro*, starting where fractal distribution ends. Allometric scaling in non-vascular spherical tissue constructs was assessed using models of Michaelis Menten oxygen consumption and diffusion. The models demonstrate that physiological scaling is maintained when about 5 to 60% of the construct is exposed to oxygen concentrations less than the Michaelis Menten constant, with a significant concentration gradient in the sphere. The results have important implications for the design of downscaled *in*-*vitro* systems with physiological relevance.

Allometric scaling laws, which correlate the mass of organisms with physiological parameters through an exponent “b”, have been explored by scientists for well over a century. Probably the best known allometric relationship is the power law describing the mass (M) and oxygen consumption or metabolic rate (MR) correlation in animals (Eq. 1). According to Kleiber^1^ and many other scientists, the exponent b for MR is ≈3/4 and the corresponding equation is known as the 3/4 power law (Eq. 1)^2^,^3^,^4^. Although the precise value of b is still debated^5^,^6^, the allometric scaling of MR and its related mass specific and cellular metabolic rate (CMR) have been the subject of numerous publications^7^,^8^,^9^,^10^. The reason behind allometric scaling of metabolic rate is still not clearly understood; nonetheless, the remarkable consistency of the so-called “quarter power laws” for metabolic rates and other metabolism related parameters over several orders of magnitude of mass have led West and Brown, widely considered as the current gurus of allometry, to deem them universal laws in biology^4^. In essence, biological organisms are assembled according to the same basic design rules and using the same building blocks (mainly water) such that self-similarity is preserved across all scales. Based on this principle, almost two decades ago West, Brown & Enquist used the fact that many organisms have fractal-like networks for resource transport to predict a value of b = 3/4 for metabolic rate^11^.

The 3/4 power allometric scaling law for MR is:


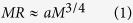


Where, *a* is a constant for all mammals, M is body mass in kg and MR is the whole body metabolic rate herein expressed in moles of oxygen consumed/s.

Given that most mammalian cells are mass invariant, cell density (ρ in #cells/m^3^) can be considered constant^12^,^13^. The density of all biological organisms is close to that of water (Ω = 1000 kg/m^3^), hence the metabolic rate per cell, or CMR in moles of oxygen/(cell.s) can be expressed as:


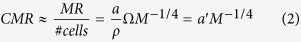


A log-log graph of CMR against mass gives a straight line with a slope of b ≈ −1/4 (Fig. 1), indicating that the oxygen consumption rate per cell increases in an organism as its body mass decreases.

It is well known that different tissues and cell types consume oxygen at different rates according to their metabolic requirements^14^ and the CMR in Eq. 2 therefore represents an average metabolic rate per cell in an organism. The CMR versus mass relationship has been explored by West and co-workers^8^. By examining CMR data from cells in culture and comparing them with whole animal CMR values (derived using eqs 1 and 2) they demonstrated that metabolic scaling is not conserved *in*-*vitro*. For all mammals, individual cells *in*-*vitro* consume oxygen at a faster rate than *in*-*vivo*. Moreover, the CMR of cultured mammalian cells converges to an approximately constant value independent of the mass of the animal of origin. They hypothesized that the number of mitochondria per cell from any mammal settles to a constant maximal value after several generations in culture. Thus, the log-log slope of *in*-*vitro* CMR versus the mass of the animal from which they derive is near zero (Fig. 1). A recent analysis of *in*-*vitro* and *in*-*vivo* oxygen consumption rates suggests that when hepatocytes are freshly isolated their CMR follows somewhat less than – 1/4 power scaling^10^. The author (Glazier) concludes that metabolic rate and its scaling with mass is not only determined by energetic or physical constraints but also by systemic regulation.

The application of allometry to the design of *in*-*vitro* systems was first proposed by Vozzi *et al*. in 2009^15^. Allometric scaling was used to engineer a “physiologically relevant” on-a-plate multi-organ model with the objective of somehow extrapolating the results to human physiology. Since then several researchers have discussed the use of allometry for scaling down human body parameters to on-chip or on-plate devices, although very few have actually implemented systems based on allometric scaling^16^,^17^. Ucciferri *et al*.^18^ show that scaling cell numbers in a 2 compartment hepatocyte and endothelial cell model can better mirror human glucose metabolism than scaling metabolic rate and surface area. However, despite current efforts to design organ and body-on-chip systems, *in*-*vitro* models of biological tissue are considered functionally inferior to whole organisms and their translational potential is still limited. In a seminal paper, Moraes *et al*.^19^ suggested that *in*-*vitro* or on-chip systems should be designed on the principle of conserving “metabolically-supported functional scaling” so as to maintain *in*-*vivo* cellular metabolic rates when down-sized. Based on this principle, physiologically meaningful *in*-*vitro* on-chip or on plate multi-organ systems should follow the quarter power allometric laws expressed in Eqs 1 and 2 as the size of devices is reduced to the microscale. One of the strategies proposed was to somehow modulate oxygen supply to cells to control their metabolic consumption.

Here the metabolic response of *in*-*vitro* constructs was characterized to determine a working window in which engineered tissues maintain allometric scaling in the absence of vascularization As cellular oxygen consumption is regulated by Michaelis Menten (MM) reaction kinetics^20^, the average MR and CMR of cell-filled spheroids were determined using computational mass transfer models coupling the MM reaction of oxygen and its diffusion through the construct.

## Theory

Assuming spherical symmetry, the reaction-diffusion equation in spherical coordinates for Michaelis Menten mediated oxygen consumption is:


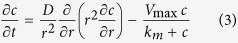


Where *c* is the oxygen concentration in moles/m^3^, D is the diffusion constant of oxygen in water (m^2^/s) at body temperature, *V*_*max*_ is the maximum oxygen consumption rate per unit volume (moles/(m^3^.s)) and *k*_*m*_ is the MM constant in moles/m^3^. Both *k*_*m*_ and *V*_*max*_ are rather difficult to measure, particularly *in*-*vivo*. Typically, *V*_*max*_ is derived indirectly from measurements of flow rates and arterial and venous oxygen concentrations in organs *in*-*vivo. In*-*vitro* it is expressed as the product ρ × CMR^20^. The constant *k*_*m*_ is estimated by fitting oxygen consumption data versus ambient oxygen concentration curves^21^.

The values of *k*_*m*_ and CMR estimated from isolated mammalian tissues and cells and reported in the literature have a narrow range (within one order of magnitude), even amongst different species^8^,^20^,^21^. On the other hand, *V*_*max*_ can be thought of as the product of enzyme affinities in mitochondria - which are approximately constant across several species^22^,^23^ - and the overall density of mitochondria in a tissue. According to West *et al*.’s extrapolations, in *in*-*vitro* conditions the mitochondrial density in cells reaches an upper limiting value of ≈300/cell^8^. Therefore, at least in *in*-*vitro* monolayer cultures, *k*_*m*_ and the limiting CMR, can be assumed as material constants related to mitochondrial enzymes. Hence, without any loss of generality and in the light of the fairly narrow range of reported oxygen consumption rates *in*-*vitro*, the values of *k*_*m*_ and limiting CMR used here are from a previous study on scaling in hepatocyte cultures^24^: respectively 7.39 × 10^−3^ moles/m^3^ and 4.8 × 10^−17^ moles/(cell.s).

Considering a representative spherical tissue or cell construct with radius *R* (in m), the overall MR is the inward flux at its surface multiplied by the total surface area.


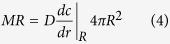


Given that a concentration gradient forms within the sphere, each cell will have a different consumption rate according to the oxygen concentration it perceives. The average CMR is then simply the MR divided by the total number of cells in the sphere.


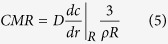


Using the above equations, the CMR was derived analytically (see Supplementary Information) or computed (see Methods) and expressed in terms of the mass of the sphere (4*πR*^3^Ω/3). Allometric relationships and estimations of “b” were determined by plotting logarithmic graphs of CMR versus mass.

## Results

As outlined in the Supplementary Information (S.I.), when the oxygen concentration is over an order of magnitude greater than *k*_*m*_ throughout the sphere (i.e. *c* >> *k*_*m*_), the reaction rate is zero order and the CMR is independent of *R* and the same for all cells (i.e. the allometric exponent for CMR is b = 0).


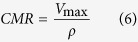


**Figure 1:**
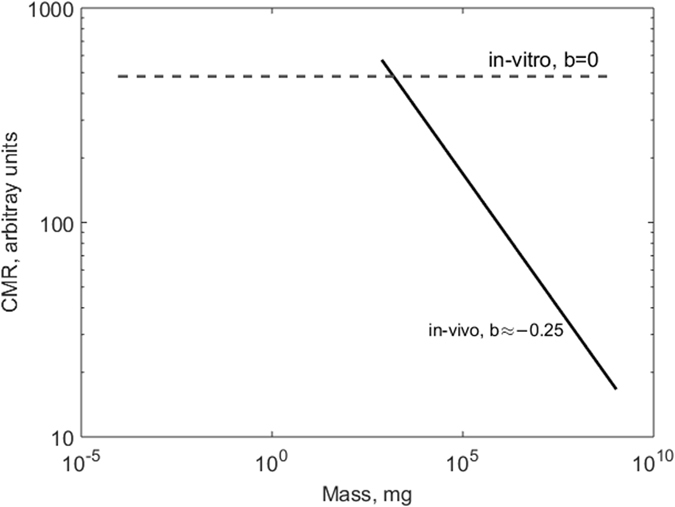
CMR as a function of the mass of an animal *in*-*vivo* and *in*-*vitro* on a log scale plot. The allometric exponent b is the slope of the line. The cellular metabolic rate increases when cells are removed from the *in*-*vivo* context and cultured *in-vitro* in the laboratory. Theoretical considerations (see text) show that this behaviour is due to high oxygen concentrations and the lack of oxygen gradients *in*-*vitro*. Figure adapted from^8^.

This very simply explains why CMR estimated *in*-*vivo* is lower than that measured *in*-*vitro*. Even in the absence of an increase in mitochondrial density *in*-*vitro*, when isolated from an organism cells are usually plated in monolayers and all perceive the same oxygen concentration. As *k*_*m*_ is typically 10 to 100 times less than the dissolved oxygen concentration in water at 37 °C in atmospheric conditions (0.2 moles/m^3^, 0.2 mM, around 20% or ≈150 mmHg)^20^,^21^, the cell oxygen consumption rate is zero order. Conversely, within an organism oxygen supply is limited and its concentration depends on the distance between cells and their arterial blood supply. Therefore, for a given organism, the average CMR *in*-*vivo* is lower than the CMR in monolayer cultures (Fig. 1).

On the other hand, when *c* << *k*_*m*_, the reaction rate is first order and the allometric exponent for CMR ranges from b = 0 to −1/3 (Fig. S3). Although this condition is unlikely *in*-*vivo* or *in*-*vitro*^25^, the theoretical analysis in the S.I. demonstrates that the CMR depends on the ratio of the reaction rate to the diffusion rate (or the Thiele modulus, *ϕ*^2^) and a quarter power metabolic scaling holds when the Thiele modulus, *ϕ*^2^ = 24.808.

**Figure 2:**
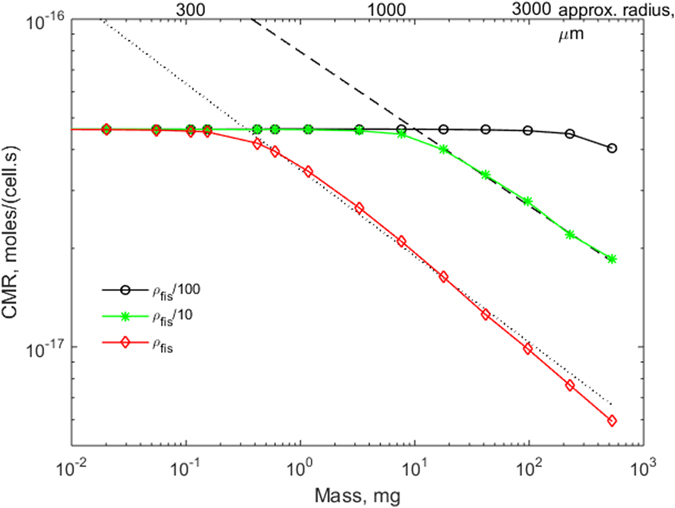
Computed CMR against construct mass for different cell densities with ambient oxygen = 0.2 moles/m^3^. Red diamonds (♢): physiological density ρ_fis_ = 5.14 × 10^14^ cells/m^3^; green asterisks (*): ρ_fis_/10, black circles (o): ρ_fis_/100. Dotted line: least squares fit with slope = −0.2631 ± 0.0222, R^2^ = 0.9946. Dashed line: least squares fit with slope = −0.2348 ± 0.0395, R^2^ = 0.9970.

In most tissues *in*-*vivo* and in some 3D (three-dimensional) cultures *in*-*vitro*, oxygen concentrations lie between *k*_*m*_ and 0.2 moles/m^3^. As Eq. 3 cannot be solved analytically for this case, to determine the conditions for– 1/4 power allometric scaling for CMR in non-vascularised *in*-*vitro* constructs, oxygen transport in 3D cell-filled spheres was computed using finite element methods. Figure 2 reports the resulting log scale graph for CMR versus mass for constructs exposed to a surface oxygen concentration (*c*_*o*_) of 0.2 moles/m^3^ with a range of cell densities from the physiological value of 5.14 × 10^14^ to 5.14 × 10^12^ cells/m^3^. The latter corresponds to about 5 million cells/mL, which is considered a fairly high *in*-*vitro* density, often used for encapsulating cells in alginate-based microspheres^26^. At low values of construct mass, the CMR does not change but tends to constant limiting value for all spheres. This is because for the smaller constructs, oxygen levels are much greater than *k*_*m*_ throughout the sphere and the reaction rate is zero order (see S.I). However, above a certain critical mass the slope becomes negative, reaching a steady value of b = −1/3 for the largest spheres with highest cell density. In fact, very large and densely packed cell constructs deplete oxygen to levels below *k*_*m*_ and as predicted by the first order reaction rate analysis reported in the S.I., the CMR scales as −1/3. The quarter power law holds in the region where the slope is around – 1/4. The window for physiological metabolic scaling was estimated by least squares fitting the linear region of the log-log plots, such that a slope of −0.25 was within the 95% confidence limits of the fitted line, with an R^2^ > 0.99.

**Figure 3:**
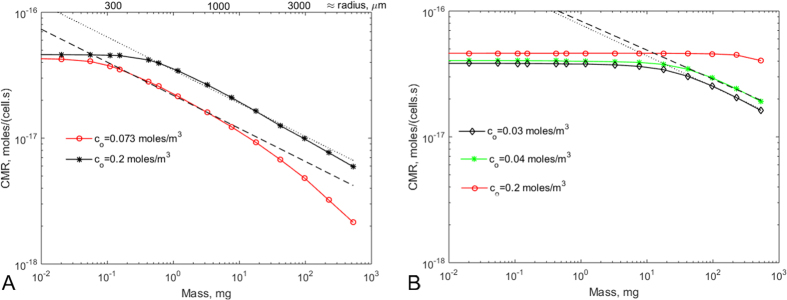
CMR versus construct mass for different ambient oxygen levels. (**A**) Physiological cell density (ρ_fis_). Black asterisks (*): dissolved oxygen in atmospheric conditions (0.2 moles/m^3^); red circles (o): O_2_ = 0.073 moles/m^3^. Dashed line: least squares fit with slope = −0.2631 ± 0.0222, R^2^ = 0.9946. Dotted line: least squares fit with slope = −0.2630 ± 0.0239, R^2^ = 0.9938. (**B**) *In*-*vitro* cell density ρ_fis_/100. Red circles (o): dissolved oxygen in atmospheric conditions, (0.2 moles/m^3^); green asterisks (*): O_2_ = 0.04 moles/m^3^); black diamonds (♢): O_2_ = 0.03 moles/m^3^. Dashed line: least squares fit with slope = −0.2309 ± 0.0579, R^2^ = 0.9932, dotted line least squares fit with slope = −0.246 ± 0.0478, R^2^ = 0.9959.

For the lowest cell density, only in the largest construct is there a notable decrease in CMR. On the other hand, the two higher cell densities have a narrow window of characteristic dimensions in which the slope closely approximates the – 1/4 necessary for physiological metabolic scaling. The spheres with physiological cell density (*ρ*_*fis*_) that fall within the quarter power window are surprisingly large considering that the maximum intercapillary distance in tissues is around 200–300 μm^27^. There is now a large body of evidence showing that tissues *in*-*vivo* thrive at low oxygen levels, certainly much lower than those routinely used *in*-*vitro*^25^,^28^. To examine the effect of ambient O_2_ concentration on the CMR, the same reaction and diffusion equation was solved for a boundary concentration of 0.0733 moles/m^3^, corresponding to 55 mmHg (rather than 150 mmHg) of O_2_ partial pressure, which is at the higher end of measured O_2_ levels in human livers^25^. The results are plotted in Fig. 3A and show how the physiological scaling region is left-shifted towards smaller constructs (down to radii of 200 to 300 μm) as oxygen concentrations are reduced to *in*-*vivo* normoxic levels. This intriguingly suggests that intercapillary volumes *in*-*vivo* are gauged so as to just tip tissues from zero to quarter power scaling and could be used as a design guideline for *in*-*vitro* constructs.

The effect of reducing ambient oxygen *in*-*vitro* to compensate for the lower cell densities usually employed in cell culture was assessed. Figure 3B illustrates how a reduction in surface oxygen levels again left shifts the window for physiological metabolic scaling towards smaller spheres.

**Figure 4:**
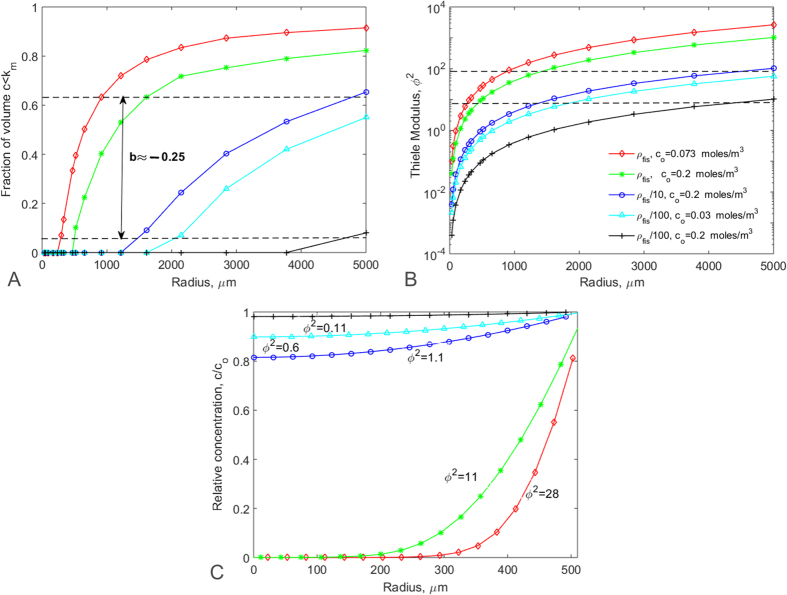
Identifying a working window for physiological scaling. (**A**) Fraction of construct volume at c < k_m_ versus construct radius showing the working window for quarter power scaling between the dashed lines. (**B**) Thiele modulus (from Eq. 7) versus radius. The quarter power window lies between the dashed lines. (**C**) Concentration gradient in 521 μm radius (≈0.6 mg) spheres with different values of cell density and ambient oxygen showing *ϕ*^2^ for each case. For this construct size, quarter power scaling was observed only for ρ_fis_ at physiological and atmospheric oxygen (i.e. *ϕ*^2^ = 11 and 28). In all cases- red diamonds (♢): physiological cell density (ρ_fis_) at physiological ambient oxygen concentrations; green asterisks (*): ρ_fis_ in ambient atmospheric oxygen; blue circles (o): ρ_fis_/10 at atmospheric O_2_; cyan triangles (Δ): ρ_fis_/100 with O_2_ = 0.03 moles/m^3^; black pluses (+): ρ_fis_/100 with atmospheric O_2_ (0.2 moles/m^3^).

Finally, to establish a set of generic quarter power scaling determinants, the fraction of construct volume with oxygen concentrations below *k*_*m*_ and the Thiele modulus (*ϕ*^2^) were plotted as a function of radius (Fig. 4A and B). Essentially, the range of masses identified in Figs 2 and 3 as falling within the window of physiological metabolic scaling correspond to spheres in which around 5 to 60% of the volume is at concentrations less than *k*_*m*_ and *ϕ*^2^ lies between ≈8 and 80. In these conditions there is a notable oxygen gradient within the construct (Fig. 4C).

## Discussion

This paper establishes a quantitative theoretical framework for estimating cellular metabolic rate (CMR) *in*-*vitro*. Using reaction-diffusion equations for oxygen transport in 3D spheriods, the analysis shows that even in the absence of vascularisation, cells and tissues can maintain power law metabolic scaling in culture under specific conditions. The model can account for any value of CMR scaling exponents between b = 0 and b = −1/3. If we suppose that the mass per cell remains constant across species, the CMR scaling exponents correspond to whole body metabolic scaling of between b = 1 (isometric scaling) and b = 2/3 (area-dependent geometric scaling). Although their investigation was not focused on allometric relationships, Milotti and co-workers also reported similar metabolic scaling exponents modelling avascular solid tumour spheroids using first order reaction kinetics and growth laws^29^,^30^.

In view of the fact that the allometric exponent for whole body metabolic scaling is widely considered to be b≈3/4 and its corresponding mass-specific exponent b = −1/4, the objective of this study was to identify the conditions for quarter power scaling of CMR in *in*-*vitro* tissues. Should other exponents be considered more appropriate, the scaling window can be shifted to higher or lower values of b. For instance, Savage *et al*. have shown that most, but not all, mammal cells are mass invariant^12^. In the few cases where cell size increases with body mass, the physiological scaling window for *in*-*vitro* constructs may need to be shifted following careful analysis of the allometric scaling parameters for the organ in question (i.e. its metabolic rate and the average cell mass in the organ).

The main experimental variables which determine whether a construct lies within a power scaling window are: its size, the cell density and the ambient or surface oxygen concentration *c*_*o*_. Specifically, “metabolically-supported functional scaling”^19^ can be maintained within a quarter power “physiological working window” wherein between 5 and 60% of the cells in the construct volume are exposed to oxygen levels below *k*_*m*_. This corresponds to a Thiele modulus of between 8 and 80 and challenges the long held presumption that tissue oxygen gradients should be minimized and *ϕ*^2^ should be close to 1^31^.

The fundamental assumptions behind the models are (i) there are no convective distribution networks inside the constructs, and oxygen transport is driven by Michaelis Menten reaction kinetics and diffusion; (ii) the oxygen levels at the surface of the constructs are constant, hence the media is well-mixed and renewed continuously, as may be the case in fluidic systems; (iii) the limiting values of CMR and *k*_*m*_ are constant, independent of construct size.

Although specific values of CMR and *k*_*m*_ were used for the computation, the results can be generalized for the fairly restricted ranges of CMR and *k*_*m*_ reported in the literature. The limiting CMR determines the absolute maximum value of the curves in Figs 2 and 3, in accordance with Eq. 6. It corresponds to the maximum consumption rate of a single isolated mammalian cell in the presence of high oxygen levels, and represents the metabolic rate to which all mammalian cells converge when cultured in standard *in*-*vitro* conditions^8^. On the bases of the considerations outlined here, it is simple to demonstrate that cells isolated from an organism and cultured in low density monolayers in media exposed to atmospheric oxygen have a higher mass specific metabolic rate (CMR) than *in*-*vivo*. On the other hand, when ambient oxygen concentrations are lower, as in some of the cases shown in Fig. 3, the maximum CMR is reduced due to lower oxygen availability (eq. S9 in the S.I.). The mass at which the slope in Figs 2 and 3 transitions from zero to negative values does depend on the Michaelis Menten constant, but the dependence is only significant when *k*_*m*_ ~*c*_*o*_, an experimentally unlikely case.

A number of reports have confirmed that the cellular oxygen consumption rate in 3D constructs is lower than in monolayers^32^,^33^,^34^. In fact, probably the simplest way to establish whether tissues *in*-*vitro* are obeying quarter power scaling is to measure tissue oxygen consumption rates as a function of construct size keeping cell density and ambient oxygen constant. Since *M* ∝ *R*^3^, should quarter power scaling hold, a 25% increase in construct dimensions will result in an ≈15% decrease in CMR. On the other hand, if the average oxygen consumption rates remain constant, then the tissue does not obey allometric scaling laws and the construct size should be increased incrementally until the CMR just begins to fall.

Very few cells in the body are ever exposed to 0.2 moles/m^3^ O_2_ under normal physiological conditions and most tissues thrive at between ≈0.1 and 0.01 moles/m^3^ 25. There is indeed a growing awareness among scientists that current *in*-*vitro* methods do not mimic the oxygen levels observed *in*-*vivo*, limiting the predictive value of cell cultures particularly as regards metabolic functions^19^,^25^,^28^. The analysis presented here suggests that the development of physiologically relevant *in*-*vitro* models with translational value requires a change in experimental paradigms as well as the development of supporting technology for monitoring and regulating oxygen. Cells should be cultured in 3D, in larger scaffolds and higher densities than previously thought acceptable and gradually coerced to re-adapt to lower oxygen concentrations and steeper concentration gradients either through proliferation and migration^33^,^35^ or a controlled reduction in ambient oxygen^36^. The results reported here have important implications for the design of more predictive and physiologically relevant fluidic devices and organ-on-a-chip systems.

## Computational Methods

To determine the allometric relationship between the CMR and mass of non-vascularised cell constructs, the reaction and diffusion of oxygen within an array of 3D cell-filled spheres was modelled using the mass transfer module in COMSOL Multiphysics (version 3.5a COMSOL AB, Stockholm, Sweden). The constants and conditions used are listed in Table 1.

The models were solved in stationary conditions using the UMFPACK direct solver. The computational grid (or mesh) was generated using the COMSOL predefined “Fine” mesh size for the 3D spheres. Once the solutions were obtained, the surface and domain integration functions were used to calculate the total inward oxygen flux at the boundaries, the average CMR and the volume of the sphere operating at *c* < *k*_*m*_. Concentration gradients were determined from domain cross section plots and the Thiele modulus was computed from^38^:


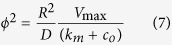


Finally, the data were imported into Matlab for plotting and curve fitting (Matlab R2015a, The Mathworks, USA. Curve Fitting Toolbox).

## Additional Information

**How to cite this article**: Ahluwalia, A. Allometric scaling in-vitro. *Sci. Rep.* **7**, 42113; doi: 10.1038/srep42113 (2017).

**Publisher's note:** Springer Nature remains neutral with regard to jurisdictional claims in published maps and institutional affiliations.

## Supplementary Material

Supplementary Information

## Figures and Tables

**Table 1:** Parameters and boundary conditions input to the computational models.

Parameter	Symbol	Range	Notes	Ref
Construct radius	R	31–5000 μm	From cell monolayers to 5 mm thick spheroids or tissue constructs.	24
Initial and boundary oxygen concentration	*c*_*o*_	0.2–0.03 moles/m^3^ or mM	Boundary conditions at the surface of the construct: implies media is well-mixed and continuously renewed at the surface.	25
Single cell CMR		4.8 × 10^−17^ moles/(cell.s)	Typical value for hepatocytes *in*-*vitro*.	20,24
Cell density	ρ	5.14 × 10^14^ −5.14 × 10^12^ cells/m^3^	From physiological cell density to typical *in*-*vitro* density (5 million cells/mL).	37
Michaeles Menten constant	*k*_*m*_	7.39 × 10^−3^ moles/m^3^	Typical value for hepatocytes.	24
Oxygen diffusion constant	*D*	3 × 10^−9^ m^2^/s	Diffusion constant in water at 37 °C.	24
Reaction rate		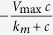	Michaelis Menten consumption.	
Stationary conditions		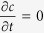		
