# Supplementary material for: Allometric scaling in-vitro
Source: Sci Rep. 2017 Feb 7;7:42113. doi: 10.1038/srep42113 (PMC5294453; doi:10.1038/srep42113)
Supplement: Supplementary Information [file srep42113-s1.pdf]

Allometric scaling in-vitro

Arti Ahluwalia

Department of Information Engineering and Research Center E.Piaggio, University of Pisa, Pisa, Italy.

*Correspondence to: arti.ahluwalia@unipi.it

Supplementary Materials

Here the CMR and its relationship with mass in a non-vascularised cell-filled spherical construct is derived from first principles in the limiting conditions of $c>>k_m$ (zero order reaction rate) and $c<<k_m$. (first order reaction rate).

The MM equation for oxygen consumption is:

$$\frac{dc}{dt}=\frac{V_{\max}c}{k_m+c} \qquad \text{(S1)}$$

The parameters and their units are given in the main text. When $c>>k_m$, oxygen consumption is zero order, that is constant and independent of *c*:

$$\frac{dc}{dt}=V_{\max} \qquad \text{(S2)}$$

When $c<<k_m$, the reaction rate depends on *c* and is first order.

$$\frac{dc}{dt}=\frac{V_{\max}}{k_m}c=Kc \qquad \text{(S3)}$$

Combining the reaction equations with diffusion of oxygen within the volume (in spherical coordinates and considering spherical symmetry) gives respectively:

$$\frac{\partial c}{\partial t}=\frac{D}{r^2}\frac{\partial}{\partial r}\left(r^2\frac{\partial c}{\partial r}\right)-V_{\max} \qquad \text{zero order}$$

$$\frac{\partial c}{\partial t}=\frac{D}{r^2}\frac{\partial}{\partial r}\left(r^2\frac{\partial c}{\partial r}\right)-\frac{V_{\max}}{k_m}c \qquad \text{first order}$$

For zero order consumption, the CMR can be easily derived for steady state conditions (Eq 5 main text) considering a constant oxygen concentration at the surface of a construct with cell density ρ.

$$CMR = \frac{V_{\max}}{\rho} \qquad \text{(S4)}$$

There is no dependence on the size of the construct and it does not follow physiological metabolic scaling. The CMR is the classic expression for the so-called oxygen consumption rate or OCR in- vitro.

In the case of first order kinetics, for a sphere with radius *R* and a constant concentration of $c=c_o$ at its surface, the equation can be solved for stationary conditions, and expressed in terms of Thiele's modulus ($\phi^2 = R^2 V_{\max} / k_m D$) giving:

$$c = c_o \frac{R}{r} \frac{\sinh\left(r\sqrt{V_{\max}/k_m D}\right)}{\sinh\left(R\sqrt{V_{\max}/k_m D}\right)} = c_o \frac{R}{r} \frac{\sinh\left(\frac{r}{R}\phi\right)}{\sinh(\phi)} \qquad \text{(S5)}$$

Again, the overall metabolic rate, *MR* in the sphere is the total inward flux at its surface multiplied by its surface area.

$$MR = D \left.\frac{dc}{dr}\right|_R 4\pi R^2 \qquad \text{(S6)}$$

Thus differentiating Eq. S5 and substituting in Eq. S6 gives :

$$MR = 4\pi c_o RD \left(\frac{\phi}{\tanh(\phi)} - 1 \right) \qquad \text{(S7)}$$

The average CMR is the MR divided by the total number of cells in the sphere ($4\pi R^3 \rho / 3$),

$$CMR = \frac{3 c_o D}{\rho R^2} \left(\frac{\phi}{\tanh(\phi)} - 1 \right) \qquad \text{(S8)}$$

Given that k_m and V_{max} can be considered scale invariant, Thiele's modulus depends on R. For spheres with small radii, i.e. ϕ^2 <<1, $\tanh(\phi)$ can be expressed as the first two terms of the series expansion for hyperbolic tangent.

$$CMR = \frac{3 c_o D}{\rho R^2} \left(\frac{\phi}{\phi - \frac{\phi^3}{3}} - 1 \right) = \frac{c_o}{\rho} \frac{V_{\max}}{k_m} \qquad \text{(S9)}$$

The CMR does not depend on the size of the construct and therefore the allometric exponent *b*=0.

On the other hand, for large tissue constructs, ϕ^2 >>1 and $\tanh(\phi)$ → 1. In this case the CMR is:

$$CMR = \frac{3c_o}{\rho R}\sqrt{\frac{V_{\max}D}{k_m}} \qquad \text{(S10)}$$

which can also be expressed in terms of the mass of the construct (water density Ω × volume) to highlight its non-physiological *b*=-1/3 allometric scaling.

$$CMR = \left[\frac{3c_o}{\rho\sqrt[3]{3/4\pi\Omega}}\sqrt{\frac{V_{\max}D}{k_m}}\right]M^{-1/3} \qquad \text{(S11)}$$

Figure S1 shows a log-log graph of CMR against the mass of a cell construct with first order consumption using the values of cell density ρ, V_{max} *and* k_m listed in Table 1 and with c_o = 0.2 moles/m^3 (atmospheric dissolved O_2). In between the two extremes of ϕ^2 which result in b=0 and b=- 1/3 respectively, for each of the curves reported in Figure S1 lies a single value of mass and Thiele's modulus where the slope equals -¼. To highlight this, Figure S2 reports the derivative of the curves in Figure S1 (i.e. b versus mass) and Figure S3 shows the derivative plotted as a function of the Thiele modulus.

To solve for the values of M and ϕ^2 which correspond to b=- ¼ , equation A8 can be expressed in terms of construct mass:

$$CMR = \frac{3Dc_o}{\rho\left(\frac{3}{4\pi\Omega}\right)^{2/3}M^{2/3}}\left[\frac{\left(\frac{3}{4\pi\Omega}\right)^{1/3}M^{1/3}\sqrt{\frac{V_{\max}D}{k_m}}}{\tanh\left(\left(\frac{3}{4\pi\Omega}\right)^{1/3}M^{1/3}\sqrt{\frac{V_{\max}D}{k_m}}\right)}-1\right] \qquad \text{(S12)}$$

and the following identity solved numerically in Matlab (using the in-built optimization functions).

$$\frac{M}{CMR}\frac{d(CMR)}{dM}+0.25=0 \qquad \text{(S13)}$$

The analysis shows that the value of M which respects quarter power scaling depends on the variables ρ,D, V_{max}, k_m but not on c_o. However, for all variables investigated and reported in Table 1, ϕ^2 =24.808 when b=- 1/4. This can be confirmed graphically from Figures S3 and S4.

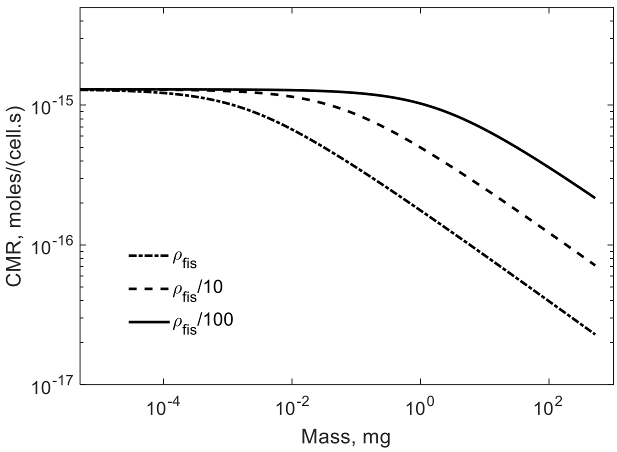

S1

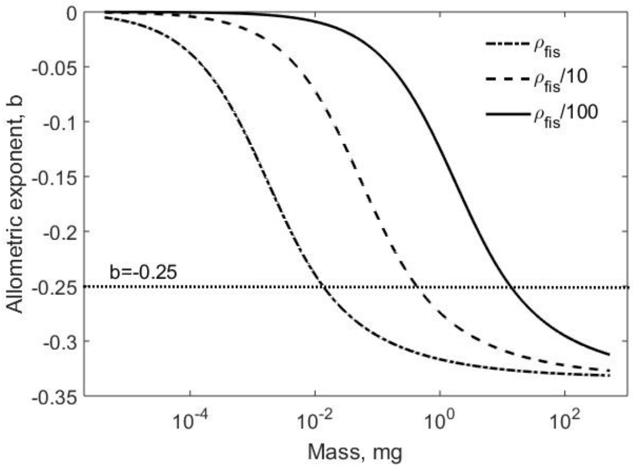

S2

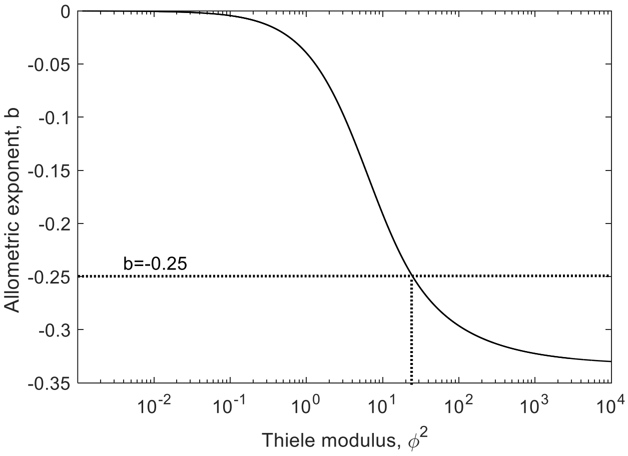

S3

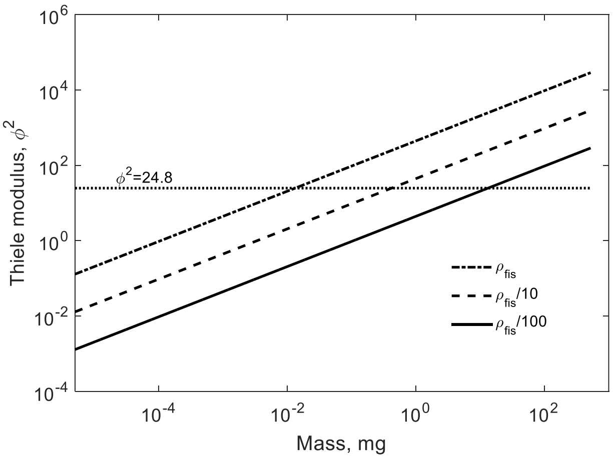

S4

Figs S1 to S4. Analysis of quarter power scaling in constructs with first order oxygen consumption. *Dotted and dashed line (.-) : physiological density ρ_{fis}=* 5.14×10^{14} cells/m^3*, dashed line (--): ρ_{fis} /10, full line: ρ_{fis}/100.* ***S1)*** *Log scale plot of CMR versus mass.* ***S2)*** *Numerical derivative of S1 (i.e. the allometric exponent b) and line corresponding to b=-1/4.* ***S3)*** *Allometric exponent b (from A2) versus Thiele modulus highlighting the unique value of ϕ^2 for which b=-1/4. The three curves for different values of ρ are coincident.* ***S4)*** *Thiele modulus versus mass. The dotted line is the value of ϕ^2 which corresponds to b=-1/4 (estimated from Figure S3 and by numerically solving the identity in equation S13).*
